# Monitoring Chemical Changes on the Surface of Kenaf Fiber during Degumming Process Using Infrared Microspectroscopy

**DOI:** 10.1038/s41598-017-01388-x

**Published:** 2017-04-27

**Authors:** Wei Jiang, Guangting Han, Yuanming Zhang, Shaoyang Liu, Chengfeng Zhou, Yan Song, Xiao Zhang, Yanzhi Xia

**Affiliations:** 1 Laboratory of New Fiber Materials and Modern Textile (The Growing Base for State Key Laboratory), Qingdao, Shandong 266071 China; 2 College of Textiles, Qingdao University, Qingdao, Shandong 266000 China; 3 Department of Chemistry and Physics, Troy University, Troy, AL 36082 USA

## Abstract

Degumming is the dominant method to obtain lignocellulosic fibers in the textile industry. Traditionally, wet chemistry methods are used to monitor the evolution of major chemical components during the degumming process. However, these methods lack the ability to provide spatial information for these heterogeneous materials. In this study, besides wet chemistry and scanning electron microscopy (SEM) analysis, a Fourier-transform infrared microspectroscopy (FTIRM) method was employed to monitor the changes in spatial distribution of the main chemical components on the kenaf surface during a steam explosion followed by chemical degum process. The results showed that hemicellulose and lignin were degummed at different rates, and the mechanisms of their degumming are different. The infrared microspectral images revealed the distribution changes of chemical components on the fiber bundle surface during the process, indicating that FTIRM is an effective tool to analyze the degumming process and improve degumming methods.

## Introduction

Natural cellulosic fiber has received more and more attention in recent years because of its sustainability, good hydroscopic property and functional properties (soft and comfort in textile industry)^1, 2^. Since most natural cellulosic fibers (except cotton) come from bast or leaf in plants, which also contain lignin, hemicellulose and other chemical components, they are often referred to as lignocellulosic fibers^3^. To obtain pure cellulose from lignocellulosic fibers, a degumming process is indispensable. Therefore, degumming is critical in the textile industry, especially in China^4, 5^. The study of degumming mechanisms and advanced degumming techniques is essential to improve the product quality and economic feasibility, as well as reduce pollution and protect the environment in the textile industry^5^.

Several degumming techniques have been carefully investigated and widely used in the last several decades, including chemical degumming (alkali, acid), biological degumming (bacteria, enzyme), physical degumming (steam explosion, ultrasonic, microwave) and their combinations^6^. The main purpose of degumming is to remove the gum matters (hemicellulose and lignin) from the surface of the fiber cell and thereafter turn the fiber cells into small fiber bundles or single fibers^7^. Most of the research focused on the chemical composition changes and the properties of the final fibers. However, since the natural fiber materials are heterogeneous, it is expected that the degumming process would also be heterogeneous. Therefore, revealing the evolution of the distributions of the major chemical components would significantly deepen our understanding of the degumming process.

Fourier-transform infrared microspectroscopy (FTIRM) is a valuable and readily available tool to identify and localize chemical components in plants^8–12^. Cell wall polysaccharides and other compounds were located in Arabidopsis thaliana petals using an FTIR microspectrometer^11^. In addition, polyhydroxybutyrate in sugarcane were successfully imaged with an FTIR microspectrometer^13^.

In the current study, kenaf fiber bundles were treated by steam explosion and chemical degumming. Wet chemistry methods and an SEM were used to investigate the changes in the fiber bundles during the degumming process. Moreover, an FTIR microspectrometer with an attenuated total reflection accessary (ATR) was employed to monitor the evolution of the distributions of cellulose, lignin and hemicellulose at microscale during the process.

## Results and Discussion

### Chemical composition analysis

Cellulose, lignin and hemicellulose are the three most important chemical components in biomass. Table 1 lists the changes of their contents at different degum stages, which provides a general idea about how the degumming process affects the chemical composition of the kenaf fiber bundle. As expected, the percentage content of cellulose increased measurably after the steam explosion and the alkali degumming treatment. Accordingly, the percentage content of hemicellulose decreased significantly during the process. The percentage content of lignin decreased as well, but at a much lower extent. The lignin content only reduced by 2.3% and 2.5% in the semi-degumming and alkali degumming processes, respectively. This is because the lignin has a more stable structure^14^, which is harder to destroy during these processes. In addition, a large portion of lignin could be inside of the fiber cells in some kinds of biomass^15^, which also prevents lignin being removed in degumming processes.

**Table 1:** Chemical compositions of raw kenaf, semi-degummed kenaf and degummed kenaf (%).

Sample	Ash	Extractives	WSM	Hemicellulose	Lignin	Cellulose
Raw kenaf	6.2 ± 0.2	1.4 ± 0.1	11.4 ± 0.3	17.5 ± 0.4	16.8 ± 0.2	54.5 ± 0.4
Semi-degummed kenaf	4.1 ± 0.3	2.6 ± 0.2	5.2 ± 0.2	11.1 ± 0.3	14.5 ± 0.2	71.1 ± 0.5
Degummed kenaf	NA	NA	NA	4.8 ± 0.4	12.0 ± 0.1	81.1 ± 0.3

Note: there is 25% weight loss after the steam explosion and additional 12% weight loss after the chemical degumming treatment. WSM represents water soluble matter.

### SEM analysis

**Figure 1:**
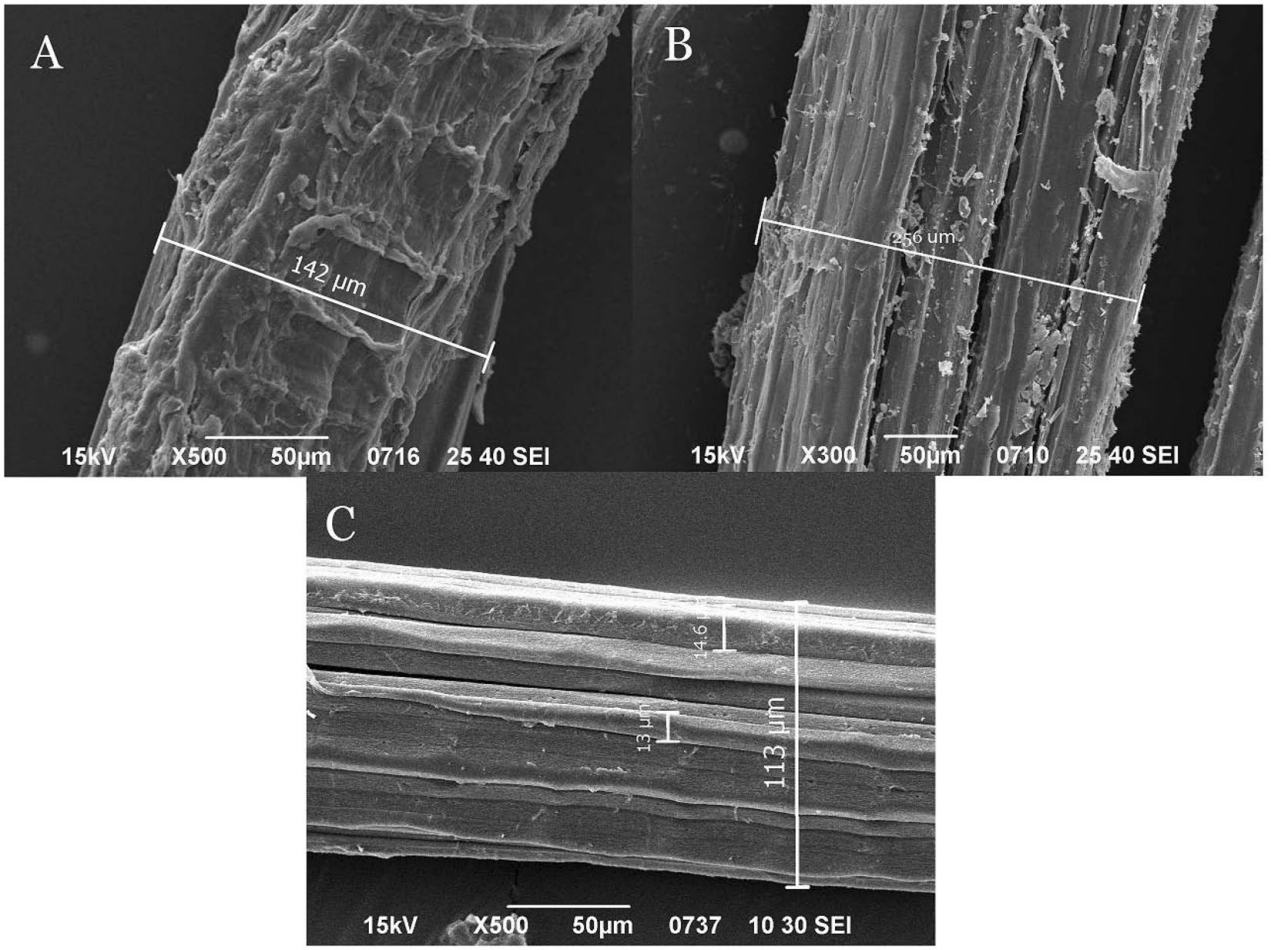
SEM image of fiber bundles (**A**) raw kenaf, (**B**) semi-degummed kenaf and (**C**) degummed kenaf.

The changes of gum on the surface of the fiber bundle are illustrated in SEM images. As shown in Fig. 1A, a raw kenaf fiber bundle was nearly completely covered by gum. After the steam explosion treatment, a large portion of the gum had been removed from the surface and some kenaf fibers were revealed (Fig. 1B). After the alkali degumming, almost all of the gum was removed from the surface of the fiber bundle and the surface became clear and smooth (Fig. 1C). The image also indicates that the gum outside the fiber cells was mostly removed during the degumming process. By comparing the masses of lignin and hemicellulose before and after the degumming (Table 1), it is suggested that about 47% lignin and 18% hemicellulose were inside of the fiber cells of kenaf. These portions of lignin and hemicellulose could not be removed by the degumming process carried out in this study. High temperature, high NaOH concentration and prolonged treatment might be necessary to remove them.

The diameters of kenaf fiber bundles and single fibers are also illustrated in Fig. 1. They are not uniform. The diameters of the fiber bundles ranges from 113 µm to 256 µm, while the single fibers have diameters around 13–15 µm.

### FTIRM analysis

Infrared microspectral analysis is a powerful tool to identify and locate chemical compounds in plants^13^. In this work, infrared microspectra were collected from raw, semi-degummed and degummed kenaf fiber bundles to monitor the evolutions of the distributions of cellulose, lignin and hemicellulose during the degumming process. The FTIRM has the ability to collect the spectra in the surface layer of the samples with a thickness of about 2 µm. This analysis would further reveal the effects of the degumming process on cellulose, hemicellulose and lignin, especially on the surface of the fiber bundle. Since the biomass is not a uniform material, it is expected that the degumming process would not be homogeneous as well. The FTIRM analysis is able to provide special distributions of these components, which would deepen our understanding of the process and guide us to improve degumming methods for different purposes.

Full IR spectra were obtained at each analysis site on the surface of the fiber bundle. Based on previous reports, cellulose, lignin and hemicellulose have evident absorption peaks at the wavenumbers of 1158 cm^−1^, 1465 cm^−1^ and 1740 cm^−1^, respectively^16^. Therefore, infrared microspectral images were generated by extracting the absorbances at these wavenumbers to represent the distributions of the corresponding components, respectively.

**Figure 2:**
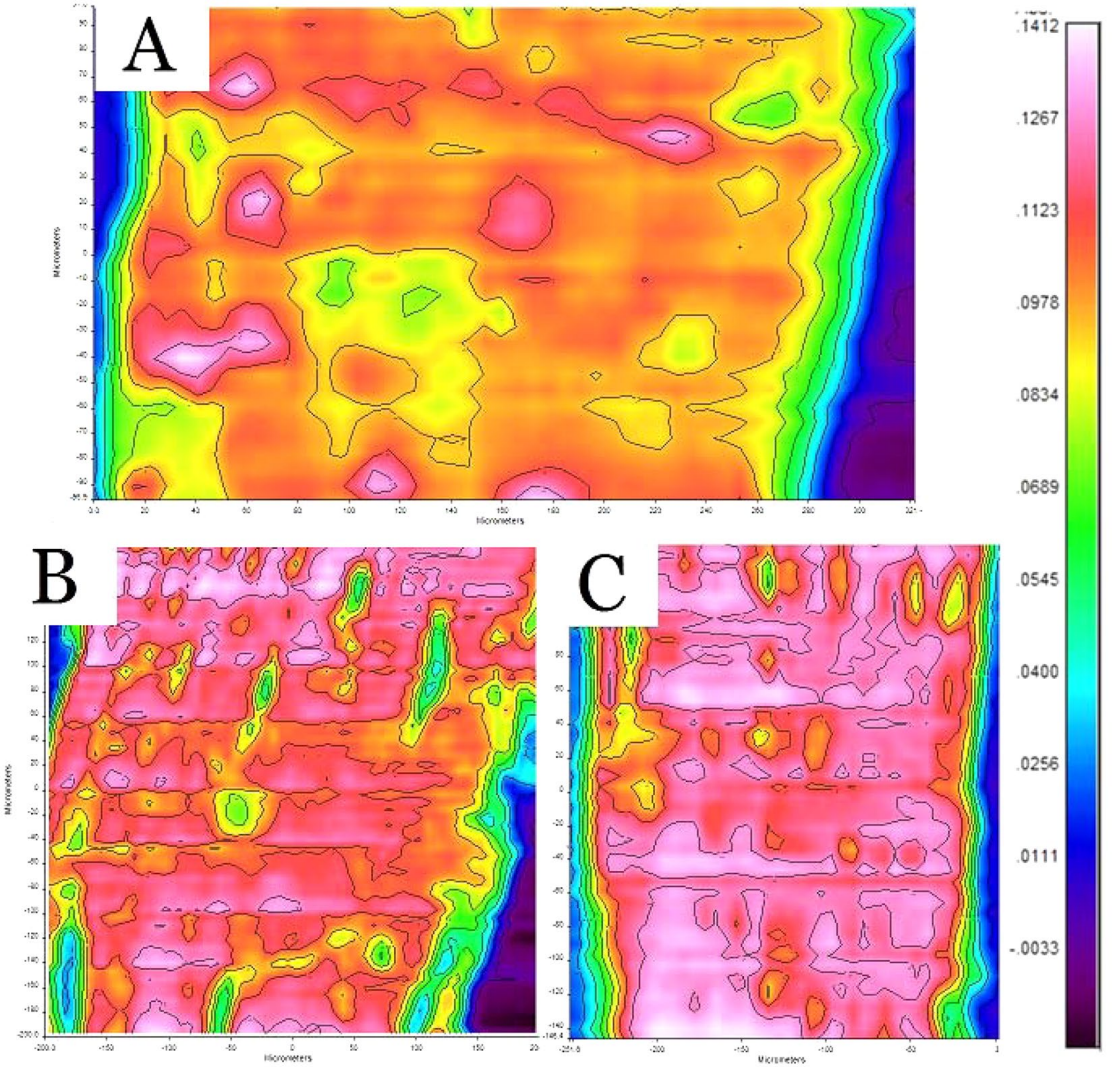
Cellulose infrared microspectral images. (**A**) raw kenaf fiber bundle, (**B**) semi-degummed fiber bundle and (**C**) degummed fiber bundle.

By choosing the wavenumber of 1158 cm^−1^, the infrared microspectral images representing cellulose distributions on the fiber bundle surface were obtained (Fig. 2). In these images, the color represents the absorption strength at the selected wavenumber, and subsequently suggests the concentration of the corresponding component in that area. The strong absorption area (pink and red) suggests that the relevant chemical component is enriched in that area on the fiber bundle surface, while the weak absorption area (yellow and green) suggests that the concentration of the corresponding component is low.

As shown in Fig. 2A, although it was mostly covered by a thin layer of lignin and/or hemicellulose, cellulose was a major component in the 2-µm thick surface layer of the raw kenaf fiber bundle. The green and yellow areas indicate a thicker covering layer of lignin and/or hemicellulose, while the red and pink areas show the locations with a thinner, or even no, covering layer. This image demonstrates that the distributions of the components are heterogeneous on the kenaf fiber bundle. After the semi-degumming (Fig. 2B) and alkali degumming (Fig. 2C), the pink and red areas expanded significantly, indicating that the lignin and/or hemicellulose were removed by the degumming process and more and more cellulose was exposed on the surface of the fiber bundle. The results are consistent with the SEM images. As listed in Table 2, the average absorption strengths of cellulose for raw kenaf, semi-degummed kenaf and degummed kenaf are 0.081, 0.087 and 0.093, respectively, confirming the trend of the change during the degumming process.

**Table 2:** Mean absorbance on the surface of kenaf fiber for cellulose, hemicellulose and lignin in different samples.

Chemical component	Wavenumber (cm^−1^)	Raw	Semi-degum	Degummed
Cellulose	1158	81 ± 5 × 10^−3^	87 ± 4 × 10^−3^	93 ± 4 × 10^−3^
Lignin	1465	67 ± 4 × 10^−3^	67 ± 4 × 10^−3^	61 ± 5 × 10^−3^
Hemicellulose	1740	29 ± 5 × 10^−3^	19 ± 5 × 10^−3^	11 ± 3 × 10^−3^

**Figure 3:**
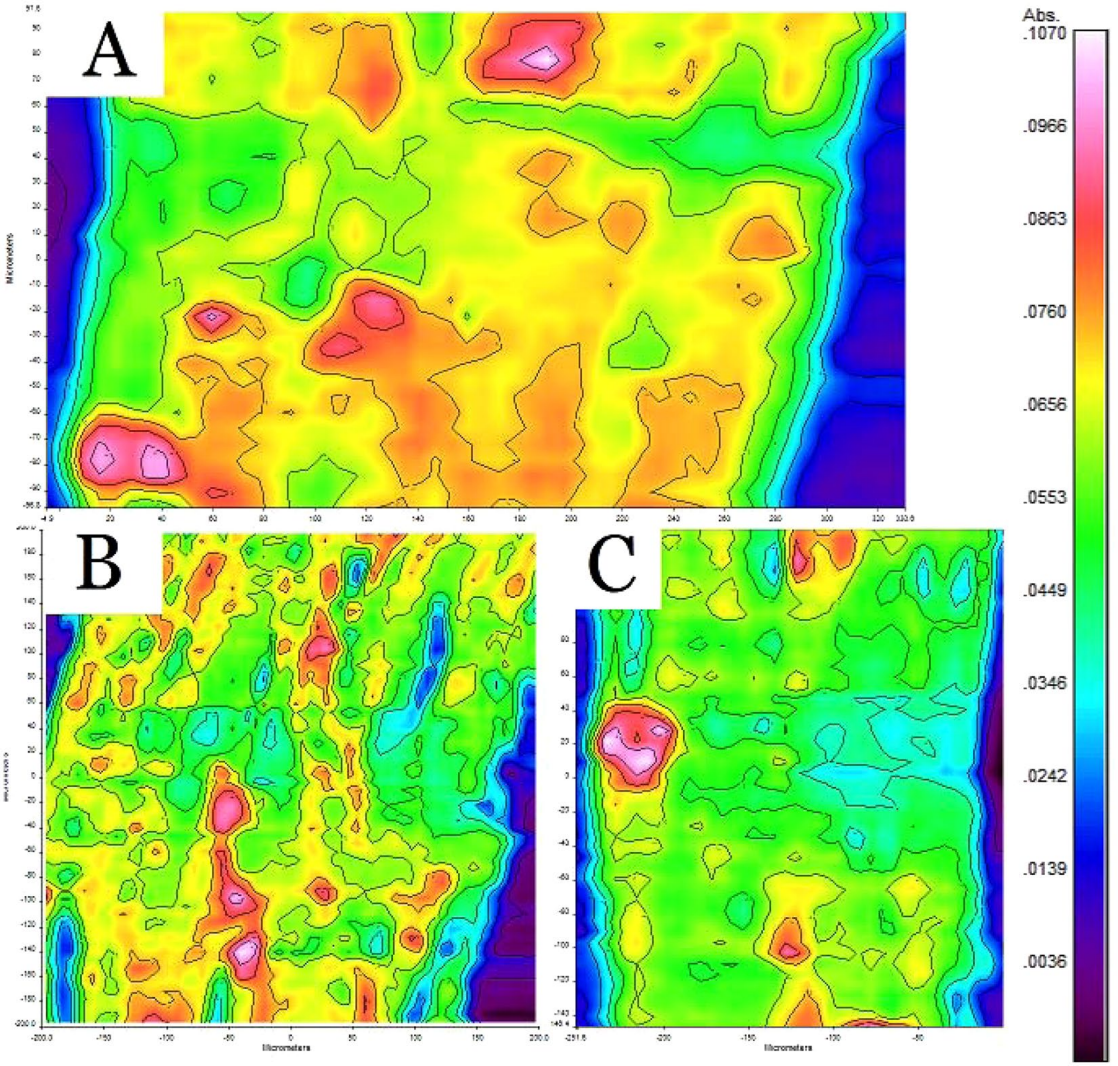
Lignin infrared microspectral images. (**A**) raw kenaf fiber bundle, (**B**) semi-degummed fiber bundle and (**C**) degummed fiber bundle.

By choosing the wavenumber of 1465 cm^−1^, the infrared microspectral images of lignin distribution were obtained (Fig. 3). A slight decrease of the lignin content, especially after the alkali degumming, was observed. The average absorption strength was changed from 0.067 to 0.061 after the second step of degumming as shown in Table 2. The images suggest that the effects of the two degumming steps on lignin removal were different. In the first step (steam explosion), the lignin was broken down to smaller particles but only a small part of lignin was removed (from Fig. 3A,B). However, in the second step (alkali boil), almost all of the broken lignin on the surface of the fiber were removed, just several big lignin particles were left (from Fig. 3B,C). In general, the lignin removal process was slow and heterogeneous, the big lignin particles were especially hard to break and remove.

Based on Figs 1C and 3C, it is noticed that most of the lignin on the fiber bundle surface had been removed during the degumming process. But, as shown in Table 1, about 47% of lignin was still in the degummed bundle, which indicates that a large portion of lignin were inside of the kenaf fiber cell. This observation explained the reason of the small lignin removal rate of the degumming process carried out in this study. To remove the lignin inside the fiber cell, stronger degumming treatment is necessary. In our future work, enzyme degumming, pre-treatment with oxidizing reagents, and/or stronger alkali degumming conditions would be investigated to degum the lignin inside kenaf fiber cell.

**Figure 4:**
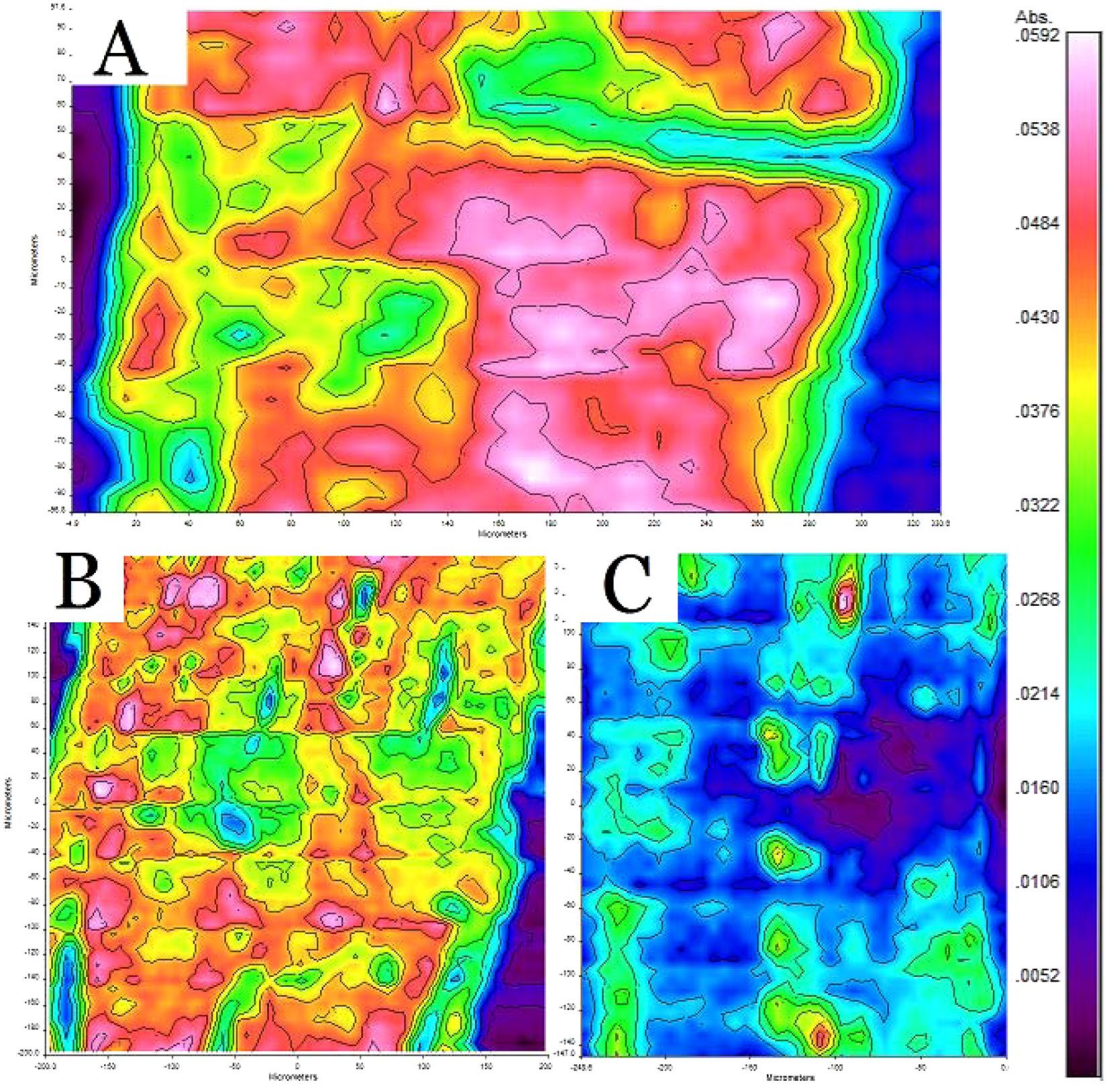
Hemicellulose infrared microspectral images. (**A**) raw kenaf fiber bundle, (**B**) semi-degummed fiber bundle and (**C**) degummed fiber bundle.

By choosing the wavenumber of 1740 cm^−1^, the infrared microspectral images of hemicellulose distribution were extracted (Fig. 4). Hemicellulose on the surface of the fiber bundle was broken down and a notable part was removed during the steam explosion. During the alkali degumming, except some small particles, almost all of the hemicellulose on the bundle surface was removed. The mean absorbance of hemicellulose was reduced from 0.029 to 0.019 in the first degumming step, and down to 0.011 in the second degumming step, confirming the high removal rate of hemicellulose of the degumming process.

## Material and Method

### Sample preparation

**Figure 5:**
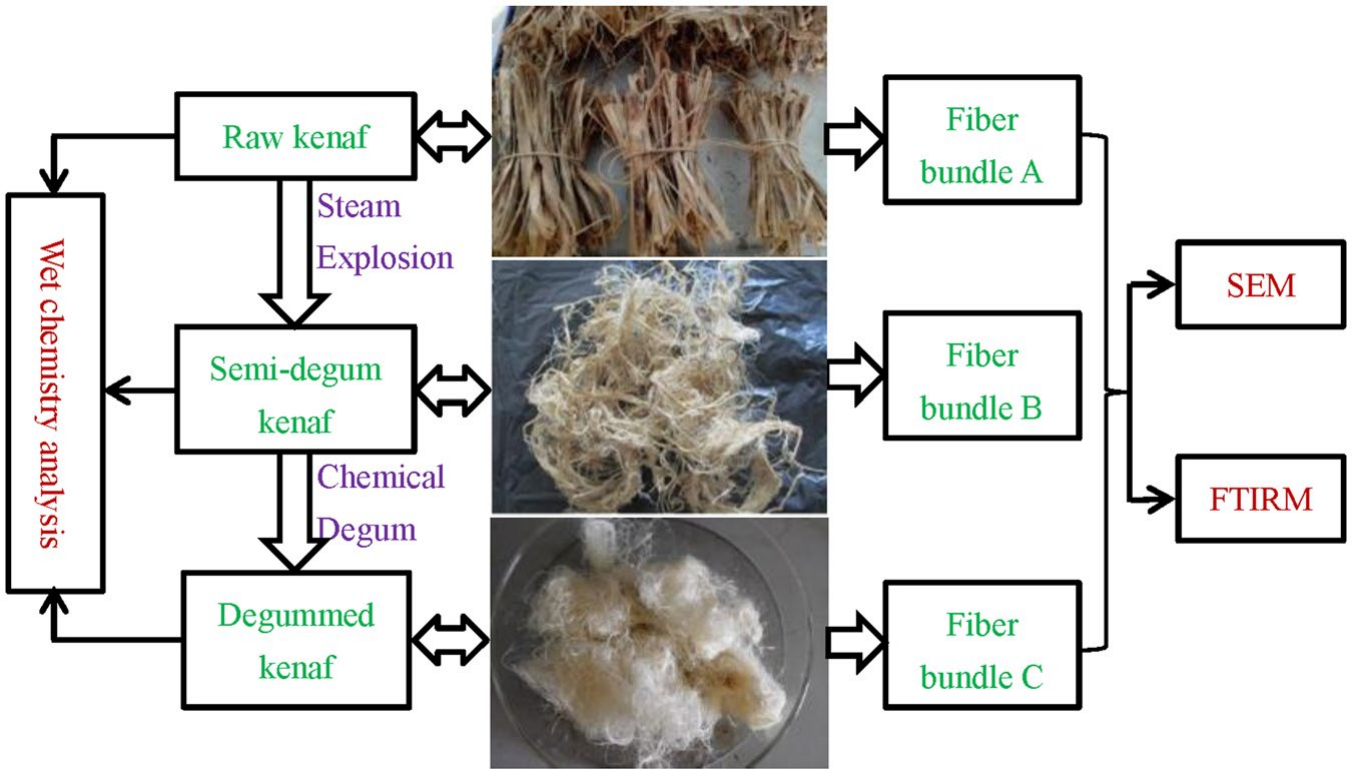
Schematic diagram for the study.

Kenaf bast samples were harvested from Xinjiang Province, China. After storage under ambient conditions for 2 weeks, 500 g of kenaf bast samples were cut to 3 cm long pieces. They were well mixed, and randomly separated into 30-g groups for the following treatments (Fig. 5).

Each group of the kenaf samples were placed in a steam explosion apparatus (QB-200, Gentle Bioenergy, Hebi City of Henan Province, China). The steam explosion treatment was carried out at 2.5 MPa for 5 min. The treatment pressure and time were optimized in our previous work^17^. After the steam explosion process, the semi-degummed kenaf was obtained.

The semi-degummed kenaf was then treated by sodium hydroxide as follows: solid to liquid ratio at 1:30, NaOH concentration at 20 g/L, H_2_O_2_ concentration at 5% (w/w), temperature at 90 °C, time at 2 h. After the treatment, the degummed fiber was dried in an oven at 105 °C for 4 h. Then, the products were sealed and stored in a desiccator until use.

Several fiber bundles from raw kenaf (kenaf bundle), semi-degummed kenaf and degummed kenaf were collected and prepared for SEM and FTIRM analyses. Each of these fiber bundle has the diameter about 100 µm.

### Wet chemistry analysis

The ash content, acid-soluble and acid-insoluble lignin contents of kenaf fibers were determined with the NREL method^18^. The extractives, water soluble matter, hemicellulose and cellulose contents were determined with the same methods as described in our previous study^17^. In brief, 150 mL benzene and ethanol (2:1 v/v) was used to extract 5 g of sample for 6 h to obtain organic extractives, then 150 mL distilled water was used to extract the residual for 3 h to get water soluble matter. The remaining extractive free sample was separated into two parts.

Part 1 was used to determine the lignin contents: A 72% (w/w) sulfuric acid treatment at 30 °C for 2 h was used to pre-hydrolyze the extractive free sample. Then, the solution was diluted to 4% sulfuric acid with distilled water, sealed in a bottle and placed in an autoclave for 1 h at 121 °C. The residual from the bottle was filtered and oven dried to measure the acid-insoluble lignin content. The solution after autoclave treatment was measured with a UV spectrophotometer at 205 nm to determine the acid-soluble lignin content. The total lignin content was the sum of the acid-insoluble lignin and the acid-soluble lignin.

Part 2 was used to determine the hemicellulose and cellulose contents: To perform the delignification, 2 g of sample were placed into a conical flask (500 mL) with 320 mL distilled water. The flask was placed in a water bath (75 °C) and then 1 mL of acetic acid and 20 mL of 15% (w/w) sodium chlorite was added every 1 h in each flask. After 4 h treatment, the residues were filtered using a filter paper, then oven dried for 3 h to measure holocellulose. After that, 1.5 g of the oven dried holocellulose was placed into a 250-mL conical flask. Then, 100 mL of 17.5% sodium hydroxide were added and the air in the flask was replaced with nitrogen. The flask was placed in a water bath at 20 °C and swilled occasionally to stir the contents. On completion of the reaction, the residues were filtered through a pre-weighed filter paper, washed with at least 500 mL distilled water, and oven dried at 105 °C overnight. The residue was determined as cellulose, the hemicellulose content was considered to be the difference between the holocellulose and the cellulose. All the experiments were conducted in triplicate.

### Scanning electron microscopy (SEM)

The SEM images of kenaf fiber bundles were acquired using a JSM 6390LV scanning electron microscope (JEOL, USA). The acceleration voltage was 1.5 kV, and the magnification was 20 to 5,000,000. Samples were stuck to the slides and coated with gold, and finally, they were investigated and photographed with the SEM.

### Fourier-transform infrared microspectroscopy (FTIRM)

FTIRM was collect with a Spotlight 400 Fourier-transform microspectrometer (PerkinElmer, USA). An attenuated total reflection (ATR) accessory was used to collect the spectra. The ATR has a 600 × 600 µm window with a resolution of 1.56 µm. The FTIRM could collect the spectrum of the components in about 2-µm thick layer at the surface of the sample. The FTIRM images were collected on 4 fibers of each sample, and 3 spots were randomly selected on each fiber to obtain an image. So, 12 images in total were acquired for each sample. The images were then analyzed with a professional FTIRM analyze software “Spectrum image”. The result of absorption strength of each image was calculated based on thousands of data points with a size of 1.56 µm × 1.56 µm in the image. In total, 12 absorption strengths of each sample were obtained. The highest absorption strength and the lowest absorption strength were excluded, and the remaining 10 results were used to calculate the average absorption strength and standard deviation for each sample.

## Conclusion

The kenaf samples were degummed with steam explosion (semi-degumming) and hot alkali (complete degumming) treatments in this work. Wet chemistry analyses suggested that hemicellulose was degummed almost completely while lignin only had a slight decrease. SEM images showed that gum on the surface of the kenaf fiber bundle was partially removed with semi-degumming process, and near completely removed after the complete degumming. Infrared microspectral images verified the above observation, and further revealed the evolutions of the distributions of cellulose, lignin and hemicellulose on the surface of the fiber bundle during the degumming process. The results demonstrated that FTIRM is an effective tool to analyze degumming process and the resulted observations could be very useful to improve degumming methods.
